# The transcriptional repressor HDAC7 promotes apoptosis and c-Myc downregulation in particular types of leukemia and lymphoma

**DOI:** 10.1038/cddis.2014.594

**Published:** 2015-02-12

**Authors:** B Barneda-Zahonero, O Collazo, A Azagra, I Fernández-Duran, J Serra-Musach, A B M M K Islam, N Vega-García, R Malatesta, M Camós, A Gómez, L Román-González, A Vidal, N López-Bigas, A Villanueva, M Esteller, M Parra

**Affiliations:** 1 Cellular Differentiation Group, Cancer Epigenetics and Biology Program (PEBC), Bellvitge Biomedical Research institute (IDIBELL), Avenida Gran Via 199, 08908 L'Hospitalet, Barcelona, Spain; 2 Breast Cancer and Systems Biology Unit, Translational Research Laboratory, Catalan Institute of Oncology (ICO), Bellvitge Institute for Biomedical Research (IDIBELL), Avenida Gran Via s/n km 2.7, 08907 L'Hospitalet, Barcelona, Spain; 3 Department of Genetic Engineering & Biotechnology, University of Dhaka, Dhaka, Bangladesh; 4 Department of Hematology, Hospital Sant Joan de Déu, Barcelona, Spain; 5 Cancer Epigenetics Group, Cancer Epigenetics and Biology Program (PEBC), Bellvitge Biomedical Research institute (IDIBELL), Avenida Gran Via 199, 08908 L'Hospitalet, Barcelona, Spain; 6 Department of Pathology, University Hospital of Bellvitge, Bellvitge Biomedical Research Institute (IDIBELL), Barcelona, Spain; 7 Research Unit on Biomedical Informatics, Department of Experimental and Health Sciences, Universitat Pompeu Fabra, Dr Aiguader 88, 08003 Barcelona, Spain; 8 Institució Catalana de Recerca i Estudis Avançats (ICREA), Barcelona, Spain; 9 Translational Research Laboratory, Catalan Institute of Oncology, Bellvitge Biomedical Research Institute, Barcelona, Spain; 10 Department of Physiological Sciences II, School of Medicine, University of Barcelona, Barcelona, Spain

## Abstract

The generation of B cells is a complex process requiring several cellular transitions, including cell commitment and differentiation. Proper transcriptional control to establish the genetic programs characteristic of each cellular stage is essential for the correct development of B lymphocytes. Deregulation of these particular transcriptional programs may result in a block in B-cell maturation, contributing to the development of hematological malignancies such as leukemia and lymphoma. However, very little is currently known about the role of transcriptional repressors in normal and aberrant B lymphopoiesis. Here we report that histone deacetylase 7 (HDAC7) is underexpressed in pro-B acute lymphoblastic leukemia (pro-B-ALL) and Burkitt lymphoma. Ectopic expression of HDAC7 induces apoptosis, leads to the downregulation of c-Myc and inhibits the oncogenic potential of cells *in vivo*, in a xenograft model. Most significantly, we have observed low levels of HDAC7 expression in B-ALL patient samples, which is correlated with the increased levels of c-Myc. From a mechanistic angle, we show that ectopically expressed HDAC7 localizes to the nucleus and interacts with the transcription factor myocyte enhancer factor C (MEF2C) and the corepressors HDAC3 and SMRT. Accordingly, both the HDAC7–MEF2C interaction domain as well as its catalytic domain are involved in the reduced cell viability induced by HDAC7. We conclude that HDAC7 has a potent anti-oncogenic effect on specific B-cell malignancies, indicating that its deregulation may contribute to the pathogenesis of the disease.

Proper generation of mature B lymphocytes is the result of complex cell lineage commitment and differentiation processes. Each cellular transition is tightly regulated at the transcriptional level by the action of linage-specific transcription factors (TFs), such as PU.1, Ikaros, myocyte enhancer factor C (MEF2C), E2A and PAX5 among others.^1, 2, 3, 4, 5, 6, 7, 8, 9^ The deregulation of these particular transcriptional programs may result in a block in the differentiation and a hyperproliferative cellular state, thereby contributing to the development of hematological malignancies such as leukemia and lymphoma. Aberrant expression or mutation of many of the lineage-specific TFs involved in B lymphocyte development have been linked to the outcome of hematopoietic malignancies.^10, 11^ In addition, the overexpression of c-Myc has been found in T-cell acute lymphoblastic leukemia (T-ALL) and B-ALL, and some types of B-cell lymphoma, such as Burkitt lymphoma, present translocations in the *MYC* gene (*c-MYC-IgH*).^12^ The deregulation of B-cell TFs in combination with chromosomal aberrations, such as gene translocations (*ETV6-RUNX1* and *BCR-ABL1*) and rearrangements in the MLL gene are key events in aberrant B lymphopoiesis and considered as primary lesions.^11, 13^

In recent years, the idea has begun to emerge that, in addition to the activation of gene expression, transcriptional repression is a fundamental mechanism to ensure proper B lymphopoiesis.^3, 14, 15^ Among the different types of transcriptional repressors, histone deacetylases (HDACs) are thought to be crucial enzymes in many physiological and pathological processes.^16, 17^ Mutation and/or aberrant expression of HDACs have often been observed in human disease, in particular cancer, making them important therapeutic targets.^18, 19^ In pathological situations where classic HDACs are overexpressed, HDAC inhibitors (HDIs) have emerged as promising therapeutic agents.^19^ However, it is worth mentioning that the contribution of HDACs to cancer could be due to mechanisms other than overexpression. In fact, HDACs may also present truncating or inactivating mutations.^18^ Therefore, we are far from fully understanding the contribution of individual HDACs to cancer.

Of the various HDACs, HDAC7 appears to be a lymphoid-specific transcriptional repressor.^20, 21, 22, 23, 24, 25, 26^ In addition to its critical role in T lymphocyte biology, we have recently reported that HDAC7 is critical in maintaining the genetic identity of B lymphocytes.^20^ Interestingly, HDAC7 has been identified as a target gene in hematopoietic cancers in a PiggyBac transposon mutagenesis screening in mice.^27^ On the basis of our recent findings, we postulated that HDAC7 might be deregulated in B-cell malignancies. Here we report the loss of HDAC7 expression in cell lines established from B-ALL and Burkitt lymphoma as well as in pro-B-ALL samples from patients. Forced expression of HDAC7 induces the apoptosis of the cells. Strikingly, the presence of HDAC7 results in the downregulation of the oncogene c-Myc. HDAC7 expression interferes with the oncogenic potential of the cells in a xenograft model. Most significantly, we have found low levels of HDAC7 expression in B-ALL samples from patients, which are associated with high levels of c-Myc. Taken together, our findings suggest that HDAC7 expression may exert an anti-oncogenic activity in particular types of B-cell malignancies and that its deregulation may contribute to the pathogenesis of B-ALL and B-cell lymphoma.

## Results

### HDAC7 is underexpressed in pro-B-ALL and B-cell lymphoma

**Figure 1:**
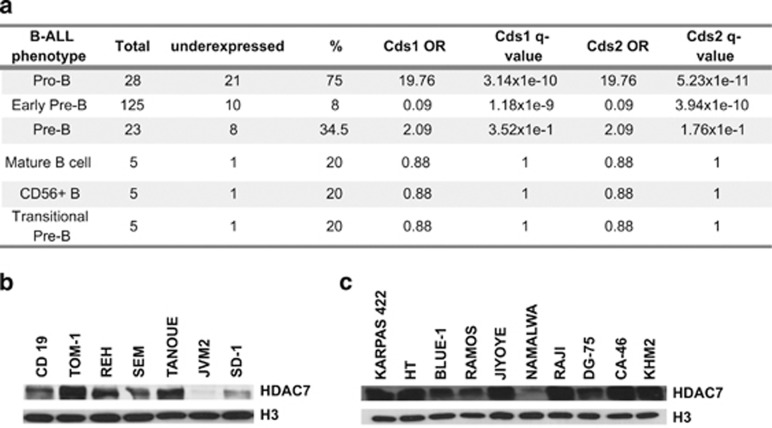
HDAC7 is underexpressed in pro-B acute lymphoblastic leukemia (ALL) and B-cell lymphoma. (**a**) Table shows the results of the Fisher test. The *q*-values result from multiple correction by FDR. The OR (odds ratio) shows the association between an ALL type and HDAC7 underexpression. OR>1 indicates that ALL type is positively associated with underexpression, whereas OR<1 indicates that it is negatively associated with underexpression. An OR=1 indicates no association between ALL type and regulation. (**b**) and (**c**) represent the western blots for HDAC7 in B-ALL and lymphoma cell lines

First, to further validate the notion that HDAC7 is a lymphoid-specific transcriptional repressor within the hematopoietic system we examined the Immunological Genome Project Database (Immgen) (http://www.immgen.org/). Using this database we confirmed that HDAC7 is specifically expressed in lymphoid cells but not in cells from the myeloid lineage (Supplementary Figure 1). This finding led us to speculate that HDAC7 expression could be deregulated in B-cell malignancies. To test this hypothesis, we first examined HDAC7 expression levels in a publicly available microarray GEO data set (GSE34861), which consists of the gene expression profile of 191 samples of adult B-ALL and 3 normal samples. The B-ALL samples comprised 28 corresponding to pro-B-ALL, 125 to early pre-B-ALL, 23 to pre-B-ALL, 5 to mature B-ALL, 5 to CD56+ B-ALL and 5 to transitional-pre-B-ALL immunophenotypes. Analysis using the Fisher test showed that HDAC7 was significantly associated with underexpression in pro-B-ALL samples (Figure 1a). We found no significant association with deregulation of HDAC7 expression in samples from the other immunophenotypes analyzed (Figure 1a). To further confirm our findings, we next assessed the HDAC7 protein levels in established cell lines from six B-ALLs. We observed that SD-1 and JVM-2 cells presented low or undetectable HDAC7 protein levels (Figure 1b). Next we tested whether HDAC7 expression could also be deregulated in lymphomas. We found that HDAC7 was underexpressed in the Burkitt lymphoma-derived Namalwa cell line (Figure 1c). Altogether, these data indicate that HDAC7 is deregulated in particular types of B-ALL and B-cell lymphoma.

### HDAC7 expression induces apoptosis in SD-1 and Namalwa cells

**Figure 2:**
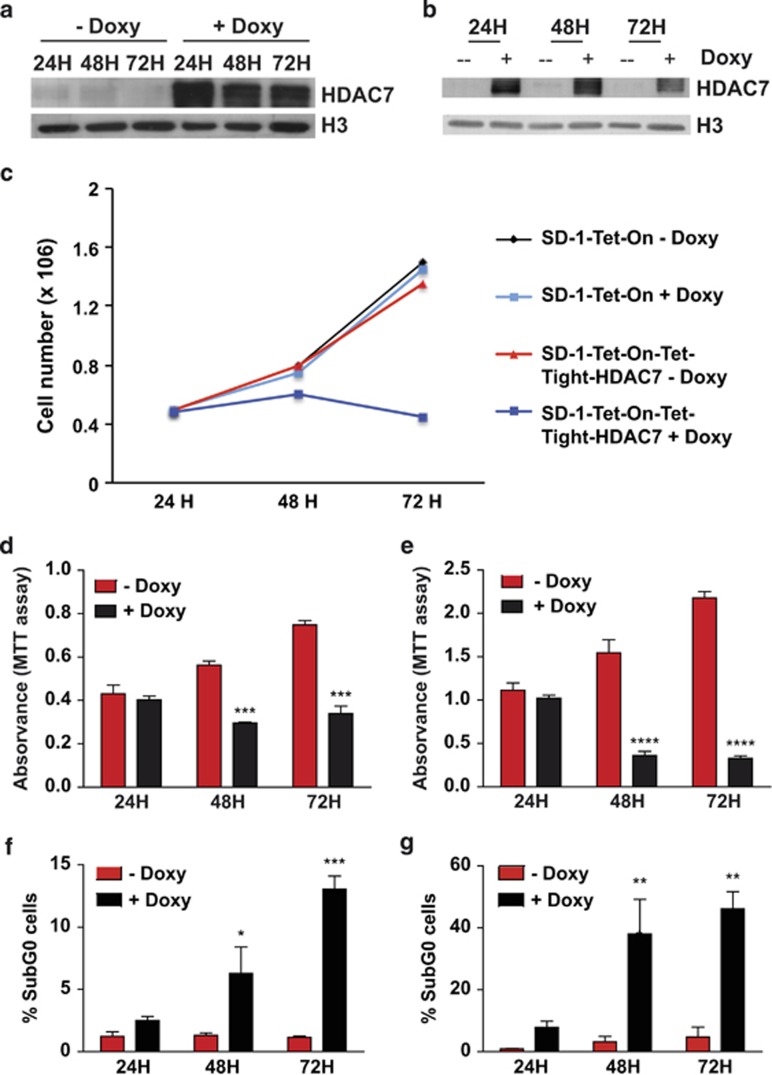
Forced expression of HDAC7 in SD-1 and Namalwa cell lines blocks their proliferation capacity and induces apoptosis. (**a**) and (**b**) SD-1 and Namalwa cells were transduced to express HDAC7 in a doxycycline-inducible manner (SD-1-Tet-On-Tight-HDAC7 and Namalwa-Tet-On-Tight-HDAC7 cells). Representative western blots showing HDAC7 protein levels after cell treatment with doxycycline. (**c**) SD-1-Tet-On and SD-1-Tet-On-Tight-HDAC7 cells were cultured and treated, or not, with doxycycline. At the indicated times after treatment, the cell number was assessed by cell counting. Trypan blue-dyed cells were omitted from the cell counts. Means±S.D. of the four independent experiments performed in triplicate. (**d**) and (**e**) Mean±S.E.M. of the absorbance units from five independent MTT assays performed in triplicate, of SD-1-Tet-On-Tight-HDAC7 (left) and Namalwa-Tet-On-Tight-HDAC7 (right) treated, or not, with doxycycline. ***P<0.001; ****P<0.0001. (**f**) and (**g**) show the percentage of cells in SubG0 from three independent experiments in SD-1-Tet-On-Tight-HDAC7 (left) and Namalwa-Tet-On-Tight-HDAC7 (right) cells treated, or not, with doxycycline for the indicated times. *P<0.05; **P<0.01; ***P<0.001

To assess whether the absence of HDAC7 is associated with the oncogenic features of SD-1 and Namalwa cells we adopted a gain-of-function experimental approach. We generated a doxycycline-inducible system to express HDAC7 exogenously in both the cell lines (Figures 2a and b). Expression of HDAC7 after the addition of doxycycline to SD-1 cells resulted in complete cell growth arrest over a course of 3 days (Figure 2c). In contrast, addition of doxycycline to the parental SD-1 cell line had no significant effect on cell growth, demonstrating that HDAC7 specifically mediates the growth arrest of the cells (Figure 2c). We then tested whether the reduction in the total number of cells was the result of a block in cell proliferation by performing an MTT assay in the absence and presence of HDAC7. HDAC7 expression significantly reduced the cell viability of both SD-1 and Namalwa cell lines (Figures 2d and e). To rule out the possibility that the effect observed on cell viability is due to the toxicity of ectopic overexpression of HDAC7, we generated HDAC7-inducible lines in cells that express normal HDAC7 levels. We found that ectopic expression of HDAC7 in RAJI and TOM-1 cell lines did not affect their viability (Supplementary Figure 2a and b). Moreover, the class IIa HDACs, HDAC4 and HDAC9 were found to be expressed in both SD-1 and Namalwa cells in the absence and in the presence of doxycycline indicating that the effect observed was specific to the expression of HDAC7 (Supplementary Figure 2c). Next we examined whether HDAC7 could induce apoptosis and assessed the cell cycle status. We observed a significant accumulation of cells in subG0 at 48 and 72 h after doxycycline treatment of both SD-1 and Namalwa cell lines, demonstrating that HDAC7 induced apoptosis (Figures 2d and e and Supplementary Figure 3). These findings indicate that the absence of HDAC7 could be associated with the survival of both the cancer cell lines and suggest that its re-expression may exert a therapeutic anti-oncogenic effect.

### HDAC7 expression induces apoptosis and inhibits tumor growth in a xenograft model

**Figure 3:**
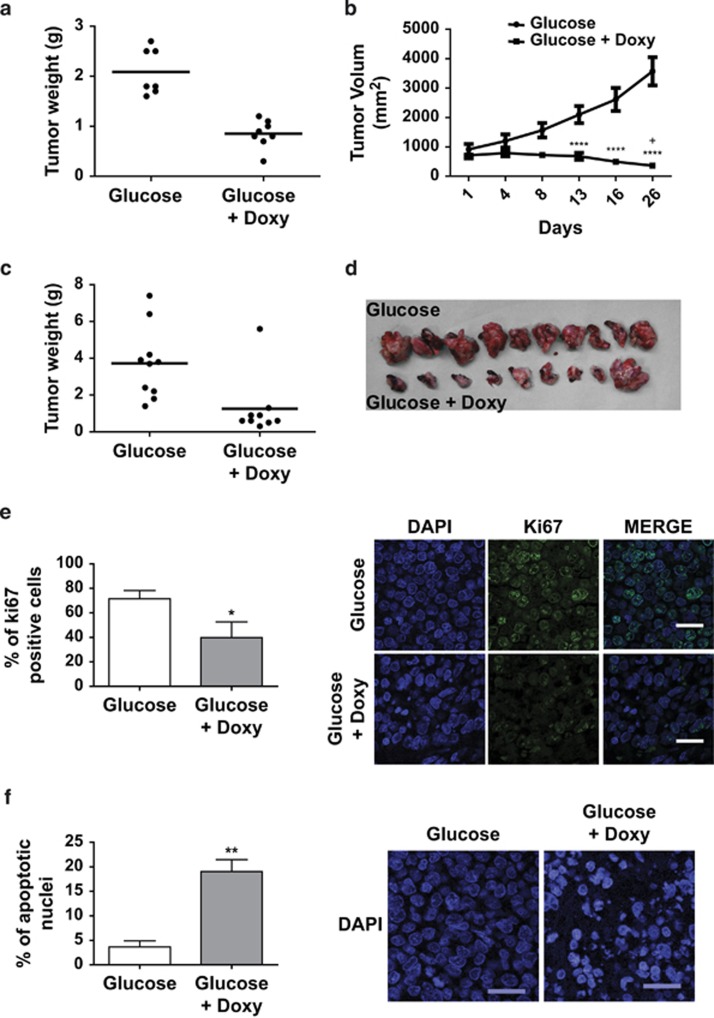
HDAC7 impairs the oncogenic capacity of SD-1 and Namalwa cells. Xenographic assays were performed with SD-1-Tet-On-Tight-HDAC7 and Namalwa-Tet-On-Tight-HDAC7 cells. SD-1 cells (5 × 10^6)^ were injected subcutaneously and 1.5 × 10^6^ Namalwa cells were orthotopically injected into the spleen. Treatment with doxycycline was started 2 weeks after injection. (**a**) and (**b**) Tumor weight at the end point of the experiment and tumor growth during the experiment of SD-1-Tet-On-Tight HDAC7 cell xenographic assays. (**c**) and (**d**) Graph of tumor weight and pictures of the tumors of Namalwa Tet-On-Tight-HDAC7 cell xenographic assays. (**e**) HDAC7 expression reduced the number of KI67-positive cells and promoted an increased in the number of apoptotic nuclei. Panel (**e**) shows the frequency of Ki67-positive cells in the SD-1-Tet-On-Tight-HDAC7 xenographic assay. More than 2400 cells per animal (three glucose; three glucose+Doxy) were analyzed. *P<0.05. (**f**) Percentage of condensed or fragmented nuclei of all nuclei from >2400 cells per animal (three glucose; three glucose+Doxy). **P<0.01

To determine the physiological consequences of HDAC7 expression in SD-1 and Namalwa cells we performed *in vivo* functional experiments using a xenograft model in athymic mice. First, SD-1 cells were injected subcutaneously into the back of several athymic mice. When tumors reached a homogeneous size they were randomly allocated into two treatment groups: (i) mice drank glucose; and (ii) drank glucose plus doxycycline in water. Tumors in mice taking glucose-treated water that did not express HDAC7 continued to grow. Strikingly, tumors in mice treated with doxycycline showed a marked decrease in their size (Figures 3a and b). Next to test the effect of HDAC7 expression on the lymphomagenic capacity of the Namalwa cell line, 1.5 × 10^6^ cells were injected orthotopically into the spleen of 19 athymic mice and they were randomly allocated into two treatment groups. (i) mice drank glucose; and (ii) drank glucose plus doxycycline in water. Notably, 15 days later, the tumors of mice treated with doxycycline were almost undetectable at palpation. At that point, all mice were killed and their spleens surgically removed. Similar to the results obtained with leukemic cells, the expression of HDAC7 in Namalwa cells markedly interfered with the growth of lymphomas (Figures 3c and d). Immunofluorescence assays revealed a significant reduction of proliferation, as revealed by Ki67 staining, and increased apoptosis in tumor cells expressing HDAC7 (Figures 3e and f and Supplementary Figure 4). Thus, our *in vivo* data confirm that HDAC7 induces apoptosis and exerts a potent anti-oncogenic effect suggesting that its absence may be involved in the pathogenesis of specific types of B-ALL and B-cell lymphoma.

### HDAC7 expression induces the apoptotic gene program of leukemic cells

**Figure 4:**
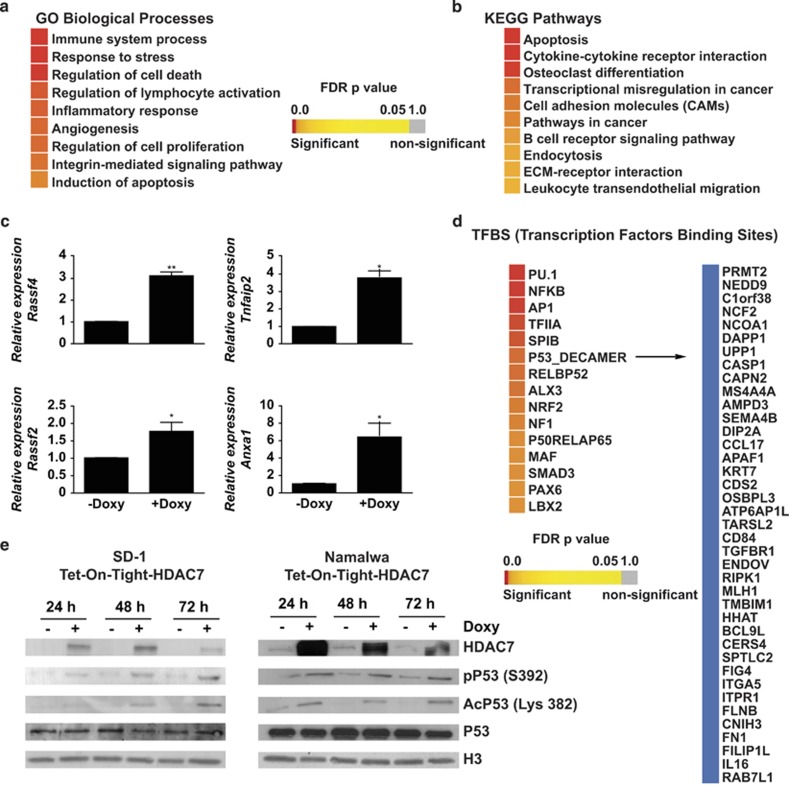
HDAC7 expression results in the enrichment of apoptotic and immune system programs and p53 activation in SD-1 cells. (**a**) and (**b**) Heatmaps showing significantly (adjusted *P*<0.05) enriched GO biological processes and KEGG pathways among the upregulated genes after HDAC7 expression. (**c**) RT-qPCR validation for the selected upregulated genes. *P<0.05; **P<0.01. (**d**) TF-binding sites enriched in the upregulated genes after HDAC7 expression. p53 target genes are shown. (**e**) Representative western blot of SD-1-1-Tet-On-Tight HDAC7 and Namalwa-1-Tet-On-Tight HDAC7 cells treated, or not, with doxycycline for the indicated times showing p53 phosphorylation and acetylation status

As HDAC7 is a transcriptional regulator, we decided to investigate the impact of HDAC7 expression on the global gene expression profile of SD-1 cells. Microarray analysis revealed that 660 genes were differentially expressed when HDAC7 was ectopically expressed in SD-1 cells. Of these, 410 genes were upregulated and 250 were downregulated (Supplementary Figure 5,Supplementary Data Sets 1 and 2). Next we examined the list of upregulated genes after HDAC7 expression and looked for the presence of apoptosis-related genes. We observed that HDAC7 induced the expression of several genes, such as *CD44*, *FAS*, *ATM*, *TP53BP2*, *CD40* and *BIRC3*, with known apoptotic functions (Supplementary Table 1). In addition, we also found that the presence of HDAC7 led to the upregulation of genes related to immune processes (*IL16*, *FCGR2A*, *IRAK2*, *CD86* and *CD40*, among others) and cancer (*RASSF4*, *RAB31*, *NEDD9* and *RASSF2*, among others; Supplementary Table 1). To further investigate if HDAC7 expression leads to the activation of the apoptotic genetic program in SD-1 cells, we performed a gene set enrichment analysis based on the gene ontology (GO) categories corresponding to the biological processes and on the KEGG pathways. The biological processes analysis revealed that the set of genes upregulated upon HDAC7 expression belong to GO categories representing immune system processes, regulation of cell death and regulation of cell proliferation, among others (Figure 4a). KEGG pathway enrichment analysis confirmed that the genes whose expression was induced by HDAC7 were significantly enriched in the apoptosis pathway (Figure 4b). A selected number of genes were validated by RT-qPCR (Figure 4c). To better understand the mechanism associated with the HDAC7-induced apoptotic pathway in SD-1 cells, we investigated the possible enrichment of TF motifs from the TRANSFAC database in the set of upregulated genes. We observed a significant enrichment of a set of TFs. Remarkably, we found the enrichment of the binding motif for p53, suggesting that the induction of apoptosis may occur in a p53-mediated manner (Figure 4d). To test this possibility we assessed the status of p53 activation after the expression of HDAC7 in both SD-1 and Namalwa cells and found that the presence of HDAC7 in both the cell lines resulted in the phosphorylation and acetylation of p53 at serine 392 and lysine 382, respectively, two post-translational modifications indicative of p53 activation (Figure 4e). Overall, our data demonstrate that HDAC7 induces apoptosis presumably via the activation of the p53 pathway.

### HDAC7 represses the expression of c-Myc

**Figure 5:**
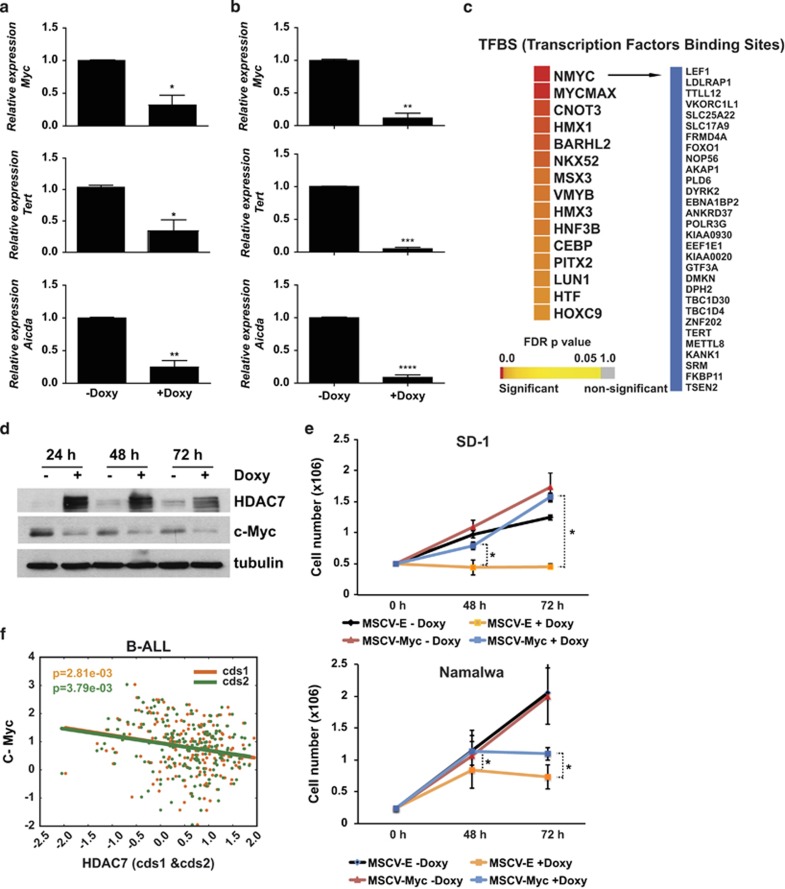
HDAC7 leads to the repression of c-Myc in SD-1 and Namalwa cells. (**a**) and (**b**) RT-qPCR validation for selected downregulated genes in the presence of HDAC7 are shown in SD-1-1-Tet-On-Tight HDAC7 (**a**) SD-1 and Namalwa-Tet-On-Tight HDAC7 cells (**b**). *P<0.05; **P<0.01; ***P<0.001; ****P<0.0001. (**c**) TF-binding sites enriched in the downregulated genes after HDAC7 expression in SD-1 cells. Myc target genes are shown. (**d**) Western blot showing the downregulation of c-Myc after HDAC7 expression in SD-1 cells. (**e**) SD-1-Tet-On-Tight-HDAC7 and Namalwa-Tet-On-Tight-HDAC7 cells transduced with either MSCV-Empty or MSCV-c-Myc retroviral vectors were cultured and treated, or not, with doxycycline. At the indicated times after treatment, the cell number was assessed by cell counting. Trypan blue-dyed cells were omitted from the cell counts. Means±S.D. of the three independent experiments performed in triplicate. *P<0.05 (**f**) GSE34861 data were analyzed to determine any correlation between *HDAC7* and *c-MYC* expression. c-MYC and HDAC7 probes were normalized with respect to values of healthy patients. The graph shows the negative correlation between c-MYC and HDAC7 (cds1 *ρ=*−2.15e-01; cds2 *ρ=*−2.09e-01) expression in the B-ALL patients

Our findings strongly indicate that HDAC7 exerts a strong anti-oncogenic effect in pro-B-ALL and B-cell lymphoma. Given that HDAC7 is a transcriptional repressor, we wondered whether its expression could lead to the repression of key oncogenes in leukemia and lymphoma. We examined our microarray data and looked for the presence of potential oncogenes in the list of downregulated genes after HDAC7 expression. Strikingly, we observed that the presence of HDAC7 resulted in the downregulation of crucial genes with known oncogenic potential, such as *MYC*, *TERT* and *AICDA* (Supplementary Table 2). This finding was validated by RT-qPCR in both SD-1 and Namalwa cells (Figures 5a and b). Using the TRANSFAC database, we found a significant enrichment of the binding site motifs for MYC factors in the HDAC7-induced downregulated genes (Figure 5c). Moreover, we also found that HDAC7 expression resulted in the reduction of c-Myc protein levels (Figure 5d). Next we tested whether the ectopic expression of c-Myc could prevent the cell growth arrest induced by HDAC7 in both SD-1 and Namalwa cells. We found that exogenous expression of c-Myc induced a significant rescue of cell growth in cells treated with doxycycline to express HDAC7. This finding further corroborates that the anti-oncogenic capacity of HDAC7 is mediated, at least in part, by the downregulation of c-Myc in SD-1 and Namalwa cells (Figure 5e). To confirm the relevance of our finding we further analyzed the published microarray GEO data set (GSE34861) and examined whether there was an association between HDAC7 and c-Myc expression levels. Strikingly, we found that a low level of expression of HDAC7 was significantly associated with high levels of c-Myc in B-ALL patients (Figure 5f). These data strongly support the hypothesis that HDAC7 posses an anti-oncogenic potential on the B-cell malignancies studied.

### HDAC7 interacts with MEF2C, HDAC3 and SMRT and is localized in the nucleus

**Figure 6:**
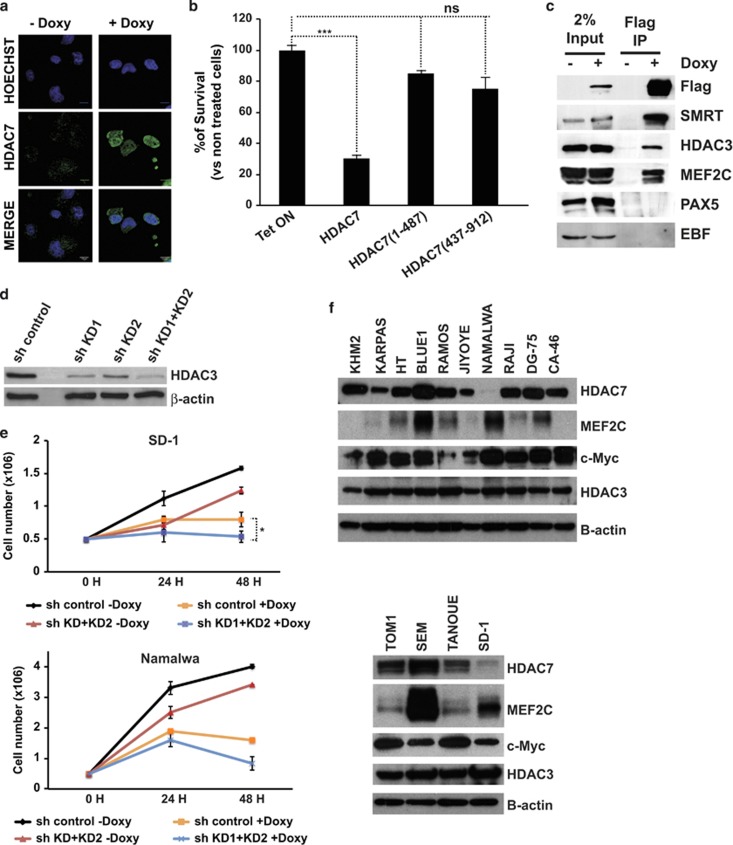
HDAC7 interacts with MEF2C, HDAC3 and SMRT and is localized in the nucleus. (**a**) Namalwa-Tet-On-Tight-HDAC7 cells were treated, or not, with doxycycline for 24 h. The subcellular localization of HDAC7 was determined by immunofluorescence. (**b**) Mean±S.E.M. of the percentage of survival cells from three independent MTT assays performed in triplicate of Namalwa cells expressing empty vector, wild-type HDAC7, and HDAC7 (1–487) and HDAC7 (438–915) deleted forms treated, or not, with doxycycline. ***P<0.001. (**c**) Cell lysates from Namalwa-Tet-On-Tight-HDAC7 treated, or not, with doxycycline were immunoprecipitated with anti-M2 agarose beads and analyzed by western blotting with the indicated antibodies. (**d**) Namalwa-Tet-On-Tight-HDAC7 cells were transduced with lentiviral vectors for the expression of the control shRNA or the shRNAs targeting HDAC3, and GFP-positive cells were purified by flow cytometry. HDAC3 protein levels were assessed by western blot. (**e**) SD-1-Tet-On-Tight-HDAC7 and Namalwa-Tet-On-Tight-HDAC7 cells transduced with either pLKO.1-shRNA control or pLKO.1-shHDAC3KD1+KD2 lentiviral vectors were cultured and treated, or not, with doxycycline. At the indicated times after treatment, the cell number was assessed by cell counting. Trypan blue-dyed cells were omitted from the cell counts. Means±S.D. of the three independent experiments performed in triplicate. (**f**) Western blots for HDAC7, MEF2C, c-Myc and HDAC3 in B-ALL and lymphoma cell lines

Class IIa HDACs posses a highly conserved C-terminal catalytic domain that mediates their recruitment to a corepressor complex containing HDAC3 and SRMT/N-CoR. In addition, class IIa HDACs contain a long N-terminal region that has been shown to mediate their interaction with tissue-specific TFs and their phosphorylation-dependent subcellular localization. To gain an insight into the mechanism of action of HDAC7, we first assessed its subcellular distribution in Namalwa cells treated, or not, with doxycycline. As expected, we found that ectopically expressed HDAC7 was mainly localized in the nuclear compartment (Figure 6a). Next we tested the potential requirement of both the TF-binding domain and the catalytic domain in HDAC7-decreased cell viability. We generated retroviral vectors carrying a C-terminal truncated construct HDAC7 (1–487) that completely lacks the HDAC catalytic domain but contains the MEF2 interacting motif, and an N-terminal truncated construct HDAC7 (438–915) bearing the enzymatic motif but lacking the MEF2 domain. Expression of wild-type HDAC7 resulted in a significant decrease in the viability of Namalwa cells, whereas the expression of HDAC7 (1–487) and HDAC7 (438–915) constructs did not have a significant effect (Figure 6b). We have recently reported that HDAC7 interacts with the TF MEF2C in B lymphocytes. To address whether ectopically expressed HDAC7 specifically interacts with MEF2C in Namalwa cells, we performed co-immunoprecipitation experiments. We found that HDAC7 associated with MEF2C and not with other B-cell-specific TFs in Namalwa cells (Figure 6c). We also observed that HDAC7 interacted with HDAC3 and SMRT (Figure 6c). It has been proposed that HDAC7 lacks any enzymatic activity and that it exerts its repressive function via the interaction with HDAC3. However, the function of HDAC7 in the absence of HDAC3 has not been properly studied. To address whether HDAC7 induces cell growth arrest through the interaction with HDAC3 in SD-1 and Namalwa cells, we performed a loss-of-function experimental approach (Figure 6d). Strikingly, we observed that HDAC3 knockdown in both cell lines reduced cell growth in the absence of HDAC7, indicating that targeting HDAC3 would also have an anti-oncogenic effect. However, in the presence of HDAC7, lowering HDAC3 levels did not lead to a rescue in the cell growth block induced by HDAC7 (Figure 6e). This result indicates that HDAC7 may possess an intrinsic enzymatic activity independent of HDAC3. Next we wondered whether the expression of HDAC7 partners could be deregulated in leukemia and lymphoma cells expressing HDAC7. To test this possibility, we determined the protein levels of HDAC3, MEF2C and c-Myc in leukemia and lymphoma cell lines. As expected, most of the cell lines express high levels of c-Myc (Figure 6f). However, we did not observed differences in the expression of HDAC3 between the cell lines tested (Figure 6f). This supports the finding that HDAC7 exerts its anti-oncogenic effect in an HDAC3-independent manner. Strikingly, we observed that in contrast to SD-1 and Namalwa cells that express the TF MEF2C, many of the other cell lines either lack or express very low levels of this TF. Therefore, the absence of MEF2C may explain why those cell lines can tolerate normal HDAC7 levels. Altogether, these experiments demonstrate that both the HDAC7–MEF2C interaction and its catalytic domain, are necessary for HDAC7 to reduce the viability of SD-1 and Namalwa cells.

## Discussion

We present data demonstrating that the transcriptional repressor HDAC7 has a potent anti-oncogenic effect in particular types of B-ALL and B-cell lymphoma. First, we report the loss of HDAC7 expression in pro-B-ALL patients and in established B-ALL and B lymphoma cell lines. Second, we show that HDAC7 expression induces apoptosis and inhibits the oncogenic potential of the cell lines tested *in vitro* and *in vivo*. Third, using genome-wide transcriptome profiling we show that ectopically expressed HDAC7 induces the expression of apoptotic genes and leads to the downregulation of key oncogenes such as c-Myc. And fourth, we report the key finding that samples from pro-B-ALL patients present low levels of HDAC7, which are associated with high levels of c-Myc expression.

The idea that HDACs are aberrantly overexpressed in cancer has been prevalent for some time, to the point where it is stated as dogma. In fact, inhibition of HDACs has been reported to have promising effects in cancer treatment.^18^ However, most HDIs are disadvantaged by their lack of enzyme specificity and have a broad range of potential side effects.^28^ Our findings from this study reveal an unexpected anti-oncogenic function for an HDAC in pro-B-ALL and B-cell lymphoma. We demonstrated that the expression of a crucial HDAC, HDAC7, for B lymphocyte biology is lost in pro-B-ALL patients and in the B-ALL and B-cell lymphoma cell lines, and that its re-expression has a potent anti-oncogenic effect. In this regard, it is important to note that underexpression levels, truncating or inactivating mutations in some HDACs in cancer have also been reported.^18^ Recently, Heideman *et al.*^29^ demonstrated that the reduction in HDAC1 and HDAC2 expression levels *in vivo* brings about T-cell lymphomagenesis owing to a block in the early thymocyte development. A different study demonstrated that the lack of HDAC3 specifically in the liver leads to the development of hepatocellular carcinomas.^30^ Therefore, our understanding of the contribution of specific HDACs to a given cancer type continues to be incomplete. Efforts are needed to establish definitively the role of specific HDACs and whether they are overexpressed, underexpressed or mutated in a particular cancer. This will allow for the design and development of HDAC isoform-specific HDIs or other molecules that can modulate the expression of a particular HDAC.

Several reports have described a potential role for HDAC7 in hematological malignancies.^27, 31, 32, 33, 34^ However, the functional contribution of HDAC7 to B-ALL remains to be elucidated. In an elegant study using a PiggyBac transposon screening in mice, Rad *et al.*^27^ revealed that HDAC7 is a target gene in hematopoietic cancers. In addition, HDAC7 has been shown to be overexpressed in childhood ALL.^34^ This discrepancy with our data could be explained by the different analytical methods used in the two studies. Tone and colleagues analyzed 94 samples from childhood ALL patients, of which 78 corresponded to B-ALL and only 4 had a pro-B-ALL immunophenotype.^34^ In the present work, we took advantage of a data set obtained in an integrative epigenomic study where they analyzed adult B-ALL patients distinguishing different immunophenotypes.^35^ Performing an accurate analysis of the expression of HDAC7, we found that HDAC7 was significantly underexpressed in pro-B-ALL patients. Therefore, it is possible that Gonzaga and colleagues did not find low levels of HDAC7 because pro-B-ALL was underrepresented in their study.

Why is HDAC7 underexpressed in specific types of B-ALL and B-cell lymphoma? Leukemogenesis and lymphomagenesis are complex malignant processes that may comprise a broad number of driver mutations, rearrangements and translocations in crucial genes, which are considered as primary lesions. We speculate that the loss of HDAC7 expression in particular types of leukemia and lymphoma may be the result of the transcriptome changes induced by a specific primary lesion. Several mechanisms could account for the loss of HDAC7 expression in leukemia and lymphoma. First, it is possible that the HDAC7 gene suffers from DNA methylation leading to its epigenetic silencing. A second potential mechanism responsible for the deregulation of HDAC7 is the action of microRNAs. In fact, another class IIa HDAC, HDAC4, has been reported to be a target of miR-155 in a Eu-miR-155 transgenic mouse model.^36^ Eu-miR-155 mice exhibit high proliferation rates of pre-B cells and develop lymphoma/leukemia. Croce and colleagues have shown that miR-155 targets HDAC4, leading to its underexpression, and that the ectopic expression of HDAC4 in diffuse large B-cell lymphoma cells inhibits miR-155-induced proliferation and increases the apoptosis of the cells.^36^ The elucidation of the molecular mechanisms involved in the repression of HDAC7 in pro-B-ALL and B-cell lymphoma is a current focus of study in our laboratory. The modulation of HDAC7 expression in specific types of B-ALL and B-cell lymphoma leading to the induction of apoptosis and the downregulation of the c-Myc oncogene may be a promising therapeutic pathway in future. On the basis of this study, HDAC7 appears to be a promising therapeutic target in these particular types of hematological disease. Our data will help to generate new, highly specific and personalized therapies for the treatment of pro-B-ALL and B-cell lymphoma.

## Materials and methods

### Plasmids and retroviral supernatant generation

pRetro-X-Tight-Pur-HDAC7 constructs were generated by cloning full length or deleted HDAC7 cDNAs obtained by EcoRI digestion of the pcDNA3.1-HDAC7 plasmids into the pRetro-X-Tight-Pur vector (Takara Bio, Otsu, Japan). MSCV-c-Myc-GFP retroviral vector was obtained from Addgene (Cambridge, MA, USA). pLKO.1-shHDAC3KD1-GFP and pLKO.1-shHDAC3KD2-GFP constructs were generated by cloning two validated shRNAs sequences that target HDAC3 (SIGMA, St. Louis, MO, USA), into the pLKO.1-GFP lentiviral vector. For retrovirus generation, pRetro-X-Tight-Pur-HDAC7 and pRetro-X-Tet-On-Advanced (Takara Bio) plasmids were transfected into the packaged cell line Platinum-E and the supernatant was collected 48 h post transfection. For lentivirus generation, the generated constructs were transfected into 293 T cells together with enveloped and packaging plasmids and supernatant was collected 48 h post transfection.

### Retroviral transduction and doxycycline treatment

Inducible HDAC7 expression in SD-1 and Namalwa cell lines was achieved by the generation of the SD-1 and Namalwa Tet-On-Tight-HDAC7 cell lines. In brief, SD-1 and Namalwa cells were first infected with the supernatant containing the pRetro-X-Tet-On-Advanced viral particles overnight and 72 h later selected with 1.5 *μ*g/ml geneticin (GIBCO, Carlsbad, CA, USA). Next the selected cells were infected with the pRetro-X-Tight-Pur-HDAC7 viral particles overnight and after 72 h selected with 3 *μ*g/ml puromycin. For HDAC7 expression, cells were treated with 500 ng/ml of doxycycline for the indicated periods. For c-Myc expression or HDAC3 knockdown, SD-1 and Namalwa Tet-On-Tight-HDAC7 cell lines were transduced with MSCV-Empty-GFP, MSCV-c-Myc-GFP, pLKO.1-GFP and pLKO.1-shHDAC3KD1+KD2-GFP and GFP-positive cells were sorted by flow cytometry.

### Proliferation and cell cycle assays

For the MTT assays, 5 × 10^4^ cells were plated onto 24-well plates. At different times, MTT was added at a final concentration of 5 mg/ml. After incubation for 3 h (37 °C, 5% CO_2_), the blue formazan derivative was solubilized in dimethyl sulfoxide and the absorbance was measured at 570 nm. Cell proliferation was also assessed by cell counting. Cell cycle and apoptosis were assessed by propidium iodide staining (distribution of cells in G_0_/G_1_, S and G_2_/M phase, and in SudG_0_) followed by flow cytometry analysis using a Gallios flow cytometer (Gallios, Beckman-Coulter, Brea, CA, USA).

### Co-Immunoprecipitation assays

Co-immunoprecipitation assays were performed as previously described in.^26^

### Mouse xenograft assay

Five-week-old male athymic *nu/nu* mice (Charles River, Wilmington, MA, USA), housed under specific pathogen-free conditions, were used in this study. To minimize tumor growth dispersion observed by subcutaneous injection of SD-1 cell line, SD-1-Tet-On-Tight-HDAC7 cells were developed in two steps: (i) 5 × 10^6^ SD-1-Tet-On-Tight-HDAC7 cells were subcutaneously injected into the back of *n*=5 animals. Once the tumors grew, they were harvested, cut into equal size small fragments and subcutaneous transplanted into the back of other nude mice. Mice bearing subcutaneous engrafted tumors (150–200 mm^3^) were randomly allocated in the two treatment groups: (i) mice drank 1% glucose; and (ii) drank 1% glucose plus 2 mg/ml doxycycline in water. The tumor growth was recorded twice per week and tumor volume (in mm3) was estimated according to the formula V=*π*/6 × L × W^2^.(W) width and (L) length. For Namalwa-Tet-On-Tight-HDAC7 cells (1.5 × 10^6^) were injected orthotopically into the middle of the spleen and the tumors were monitored by palpation twice a week. Likewise, mice were randomly allocated in the two treatment groups: (i) mice drank 1% glucose; and (ii) drank 1% glucose plus 2 mg/ml doxycycline in water. At the time of killing (30 days after induction in SD-1 cells and 15 days for Namalwa cells) all the tumors were excised and weighed, analyzed macroscopically and by hematoxylin and eosin tissue staining for histological assessment. All experiments were approved by the IDIBELL animal care and use committee.

### Immunofluorescence

Tumors were fixed in 4% formaldehyde overnight at 4 °C, embedded in paraffin wax and sectioned at 4 *μ*m. For immunofluorescence staining, antigen retrieval was performed in 10 mM sodium citrate (pH 6.0). Tumor sections were blocked with 5% horse serum in phosphate-buffered saline for 1 h at room temperature and incubated with primary antibodies overnight at 4 °C. The primary antibody used was anti-Ki67 (Thermo Scientific, Alcobendas, Spain). Tumor sections were then incubated with secondary antibodies for 1 h at room temperature. Nuclei were stained using 4′,6-diamidino-2-phenylindole. Samples were imaged on a Leica TCS SP5 spectral confocal microscope (LEICA, Barcelona, Spain), with a 63XNA 1.4 objective and using the LASAF software version 7 (LEICA). Microphotographs were analyzed with Fiji software (http://fiji.sc/). In brief, images were analyzed in gray scale and median filtered. More than 2400 cells per animal (three glucose; three glucose+Doxy) where analyzed in Ki67 staining and apoptotic nuclei.

### Western blot

Western blot analysis was performed according to standard procedures. Western blots were developed with the ECL detection kit (Amersham Biosciences, Pittsburgh, PA, USA).

### RT-qPCR expression analyses

RT-qPCR analysis were performed as previously described in.^20^

### GSE34861 analysis

Expression profiling (microarray) of 191 samples of adult B-lineage ALL and 3 normal pre-B samples were extracted from GEO database (GSE34861). Raw data were robust multichip average (RMA) normalized using the RMA algorithm in NimbleScan 2.5 software (Roche NimbleGen, Inc, Basel, Switzerland). For differential expression analysis, average Log2 expression of the normal samples were subtracted from each cancer patient expression data (Log2 value) for each gene using in house python script. The association between the immunophenotypes of the various B-ALL patients and the downregulated levels of HDAC7 was examined using the Fisher test. Two HDAC7 probes were analyzed: cds1 Homo sapiens histone deacetylase 7A (HDAC7A), transcript variant 1, mR and cds2 Homo sapiens histone deacetylase 7A, mRNA (cDNA_clone_MGC:74915_IMAGE:6179239), complete cds. Both the probes were normalized with respect to values of healthy patients. GSE34861 data were analyzed under R statistical language. The *q*-values where obtained from multiple correction by false discovery rate (FDR). The odds ratio (OR) shows the association between ALL immunophenotype and the downregulation of HDAC7. If an OR>1 indicates that ALL immunophenotype is positively associated with having downregulated levels of HAC7, whereas OR<1 indicates negative association between having the specific ALL immunophenotype and HDAC7 downregulation. An OR=1 indicates no association between ALL immunophenotype and HDAC7 expression. To identify the associations between the characterized cytogenetic features of B-ALL and low levels of HDAC7 expression we applied the Wilcox test. Data were analyzed using the R statistical language. To assess the correlation between *c-MYC* and the two *HDAC7* probes in the 191 B-ALL patients the Pearson correlation coefficient was computed (rho) and linear regression was performed.

### Microarray experiments

SD-1 Tet-On-Tight-HDAC7 or SD-1 cells were treated or not for 24 h with doxycycline and collected in Trizol reagent (Invitrogen, Carlsbad, CA, USA). RNA was extracted as described above. PCR-amplified RNAs were hybridized against an Affymetrix human array chip (Affymetrix Human Genome U219 Strip, Santa Clara, CA, USA) at the IRB genomics facility. Affymetrix raw CEL files have been deposited in the GEO database (GSE51895).

### Microarray analysis

Expression data were analyzed using the R statistical program. The RMA method was applied to the raw data. This comprises three steps: convolution background adjustment, probe-level quantile normalization and median polish summarization. Linear model analysis (LIMMA package, Bioconductor) was used to identify the significant upregulated and downregulated genes. A FDR multiple test was applied to the *P*-values obtained from the LIMMA procedure; the upregulated and downregulated genes were considered to be significant for the values of adjusted *P*≤0.05. Values of log2FC≥0.5 and log2FC≤0.5, respectively, indicated the upregulated and downregulated genes.

### Functional and pathway enrichment analysis

Functional annotation of differentially expressed genes was based on GO (Consortium, 2000; http://www.geneontology.org) as extracted from the EnsEMBL and the KEGG pathway databases. Accordingly, all genes were classified into ontology categories. We took only the GO/pathway categories that had at least 10 annotated genes. We used GiTools for enrichment analysis and heatmap generation ^37^(www.gitools.org). The resulting *P*-values were adjusted for multiple testing using Benjamin and Hochberg's FDR method.

### Putative TF motif occurrence in promoter region

The possible occurrence of the TF motif in the promoter region (500-bp upstream and 200-bp downstream of the transcription start site) was predicted with the STORM algorithm,^38^ applying a cutoff of *P*=0.0000125 and position frequency matrices from the TRANSFAC database^39^ (professional version release 2009.4).

### Statistical analyses

All data, except those from the arrays, were analyzed using GraphPad Prism5 (GraphPad, San Diego, CA, USA). Student's *t*-test and one- or two-way ANOVA, incorporating Bonferroni multiple comparisons, were carried out to evaluate the differences between the groups.

## Abbreviations

HDAC7: histone deacetylase 7
B-ALL: B-cell acute lymphoblastic leukemia
MEF2C: myocyte enhancer factor C
HDIs: histone deacetylase inhibitors
