# Supplementary material for: The transcriptional repressor HDAC7 promotes apoptosis and c-Myc downregulation in particular types of leukemia and lymphoma
Source: Cell Death Dis. 2015 Feb 12;6(2):e1635–. doi: 10.1038/cddis.2014.594 (PMC4669785; doi:10.1038/cddis.2014.594)
Supplement: Supplementary Information [file cddis2014594x1.pdf]

Supplementary Figure legends

Supplementary Figure 1. HDAC7 is a specifically expressed in lymphoid cells. HDAC7 expression levels in murine hematopoietic cell populations obtained from the Immgen public database. Numbers in the x axis represent post-normalized gene expression values. The labels shown in the y axis represent different immune cellular subtypes. A precise description of each cell type and how they were purified is provided in Immgen.org.

Supplementary Figure 2. HDAC7 expression blocks SD-1 cell growth. (**a**) and (**b**) Tom-1 and Raji cells were transduced to express HDAC7 in a doxycycline-inducible manner. Representative western blots showing HDAC7 protein levels after cell treatment with doxycycline. Mean ± SEM of the absorbance units from 3 independent MTT assays performed in triplicate. (**c**) Western blots showing HDAC4 and HDAC9 protein levels in SD-1-Tet-On-Tight-HDAC7 and Namalwa-Tet-On-Tight-HDAC7 cells treated or not with doxycycline.

Supplementary Figure 3. Forced expression of HDAC7 in SD-1 and Namalwa cell lines blocks their proliferation capacity and induces apoptosis. (**a**) Cell cycle and apoptosis were assessed by PI-staining. Distribution of the cells in G0/G1, S, G2/M phases and subG0 was analyzed by flow cytometry. Representative histograms at the indicated times after doxycycline SD-1-Tet-On-Tight-HDAC7 cell treatment are shown. cells in G1, S, G2/M phases and subG0 was analyzed by flow cytometry. Representative histograms at the indicated times after doxycycline SD-1 cell treatment are shown. (**b**) and (**c**) show the percentage of cells in G0/G1, S, G2/M phases from 3 independent experiments with (**b**) SD-1 and (**c**) Namalwa cells treated or not with doxycycline for the indicated times. * $p < 0.05$; ** $p < 0.01$.

Supplementary Figure 4. HDAC7 expression reduced the number of KI67-positive cells and promoted an increase in the number of apoptotic nuclei. Panel (**a**) shows the percentage of cleaved caspase 3 positive cells and panel (**b**) shows the frequency of Ki67-positive cells in the Namalwa-Tet-On-Tight-HDAC7 xenographic assay. More than 2400 cells per animal (3 glucose; 3 glucose + Doxy) were analyzed. * $p < 0.05$. (**c**) Percentage of condensed or fragmented nuclei of all nuclei from > 2400 cells per animal (3 glucose; 3 glucose + Doxy). ** $p < 0.01$. Fluorescence photomicrographs of representative fields are shown. Scale bar: 25 μm.

Supplementary Figure 5. Heatmap and clustering analysis of the differentially expressed genes. Heatmap and clustering analysis of the differentially expressed genes is shown.

Supplementary Datasets

Supplementary Dataset 1. List of upregulated genes after HDAC7 expression in SD-1 cells.

Supplementary Dataset 2. List of downregulated genes after HDAC7 expression in SD-1 cells.

Supplementary Tables

Supplementary Table 1. HDAC7-induced genes belonging to apoptosis, immune processes and cancer categories. SD-1 Tet-On-Tight-HDAC7 or SD-1 cells were treated or not for 24 hours with doxycycline and RNA was collected. Samples were subjected to the Affymetrix Human Genome U219 Strip array. Expression data were analyzed using the R statistical language. The robust multichip average (RMA) method was applied to the raw data. The LIMMA package was used to identify informative

upregulated and downregulated genes, $p < 0.005$. An FDR multiple test was applied to the p-values obtained by the LIMMA procedure; upregulated and downregulated genes were considered to be informative for values of adjusted $p < 0.05$. Distribution of enriched genes upregulated in apoptosis, immune system and/or cancer biological categories (FC, n-fold change *vs.* control).

Supplementary Table 2. HDAC7-repressed genes belonging to immune processes and cancer categories. SD-1 Tet-On-Tight-HDAC7 or SD-1 cells were treated or not for 24 hours with doxycycline and RNA was collected. Samples were submitted to the Affymetrix Human Genome U219 Strip array. Expression data were analyzed using the R statistical language. The robust multichip average (RMA) method was applied to raw data. The LIMMA package was used to identify informative upregulated and downregulated genes. An FDR multiple test was applied to the p-values obtained using LIMMA procedure; upregulated and downregulated genes were considered to be informative for values of adjusted $p < 0.05$ Distribution of enriched genes downregulated in apoptosis, immune system and/or cancer biological categories (FC, n-fold change *vs.* control).
