# Supplementary figures and images for: The transcriptional repressor HDAC7 promotes apoptosis and c-Myc downregulation in particular types of leukemia and lymphoma
Source: Cell Death Dis. 2015 Feb 12;6(2):e1635–. doi: 10.1038/cddis.2014.594 (PMC4669785; doi:10.1038/cddis.2014.594)

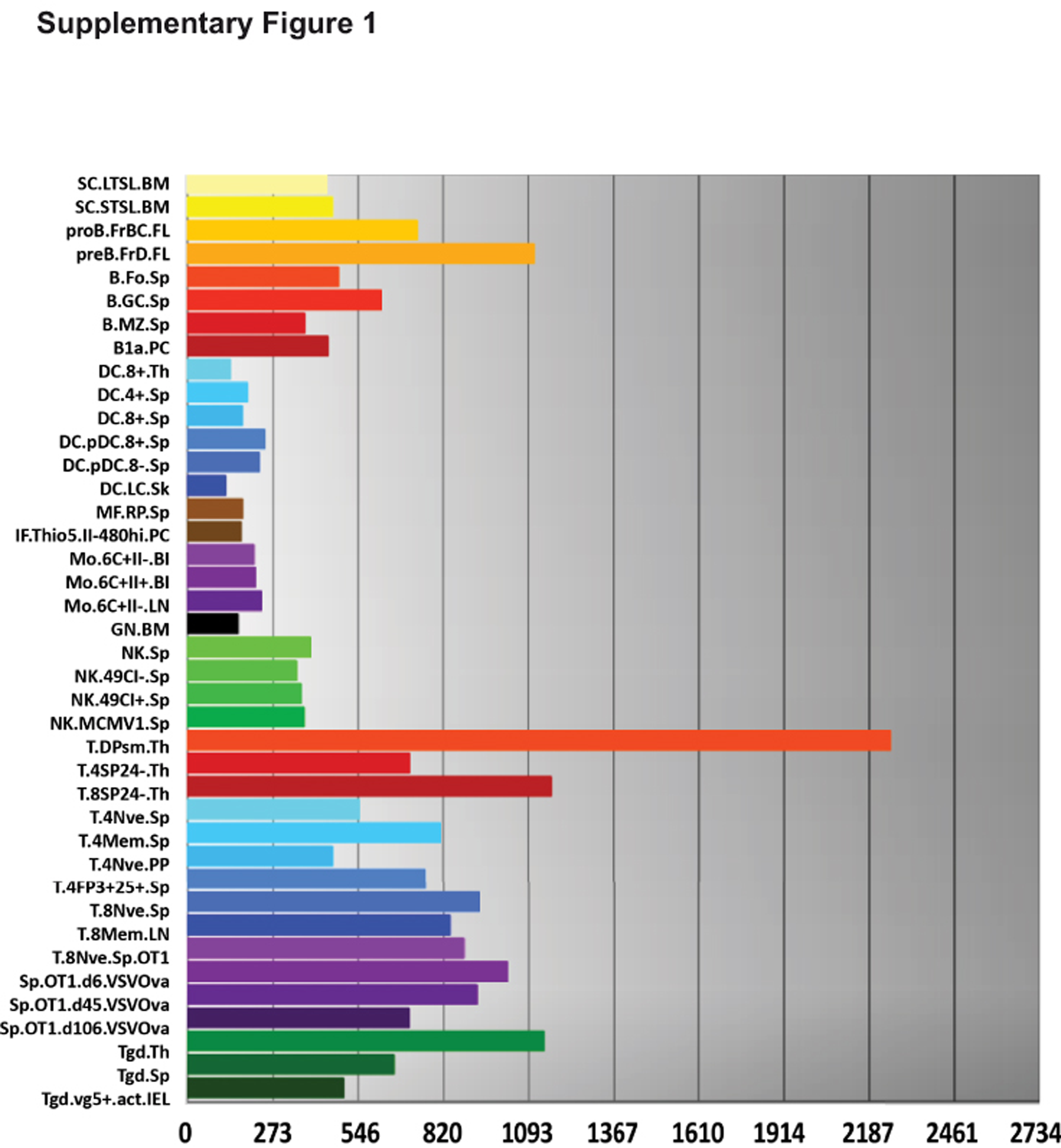

Supplement: Supplementary Figure S1 [file cddis2014594x2.tif]

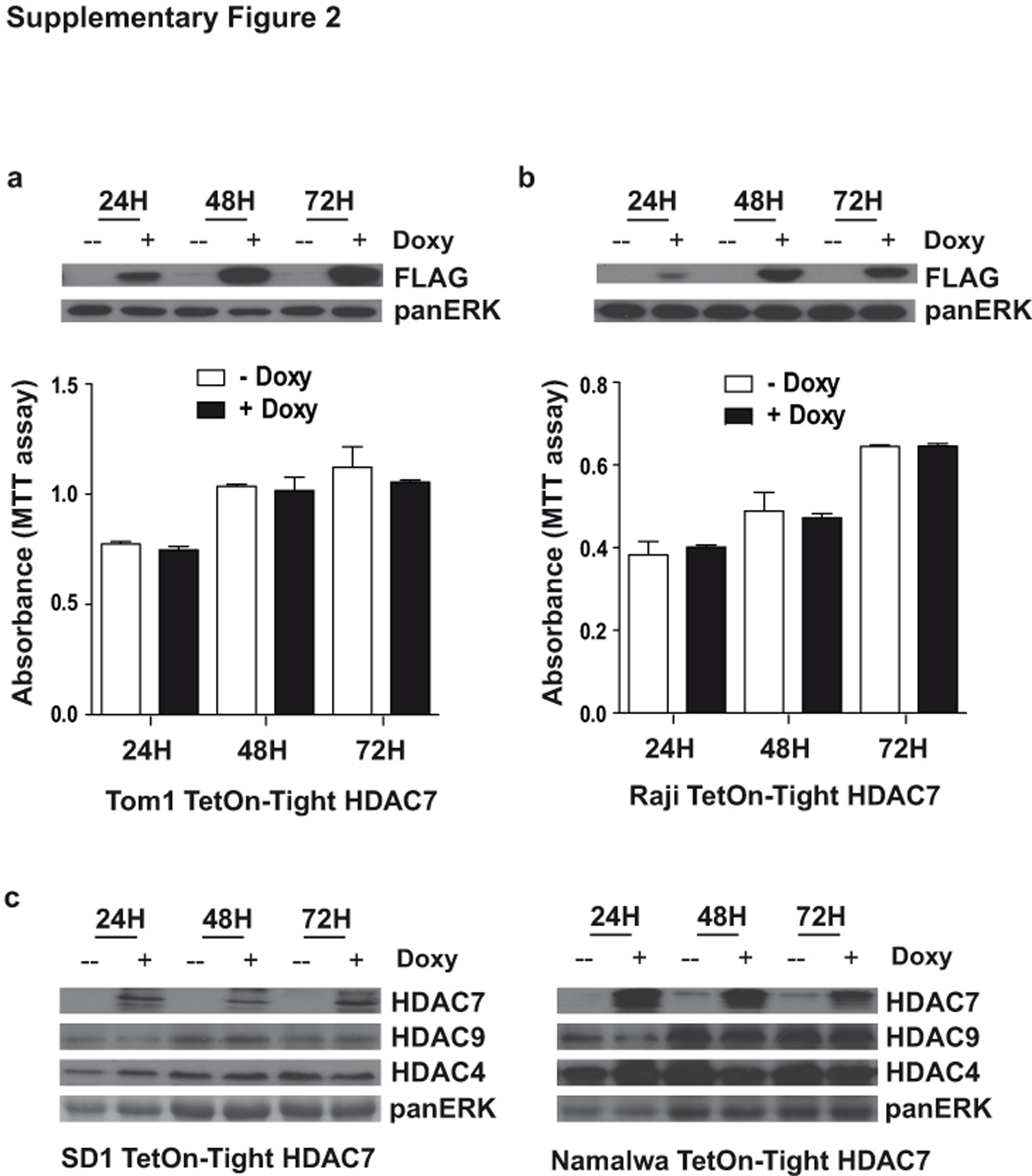

Supplement: Supplementary Figure S2 [file cddis2014594x3.tif]

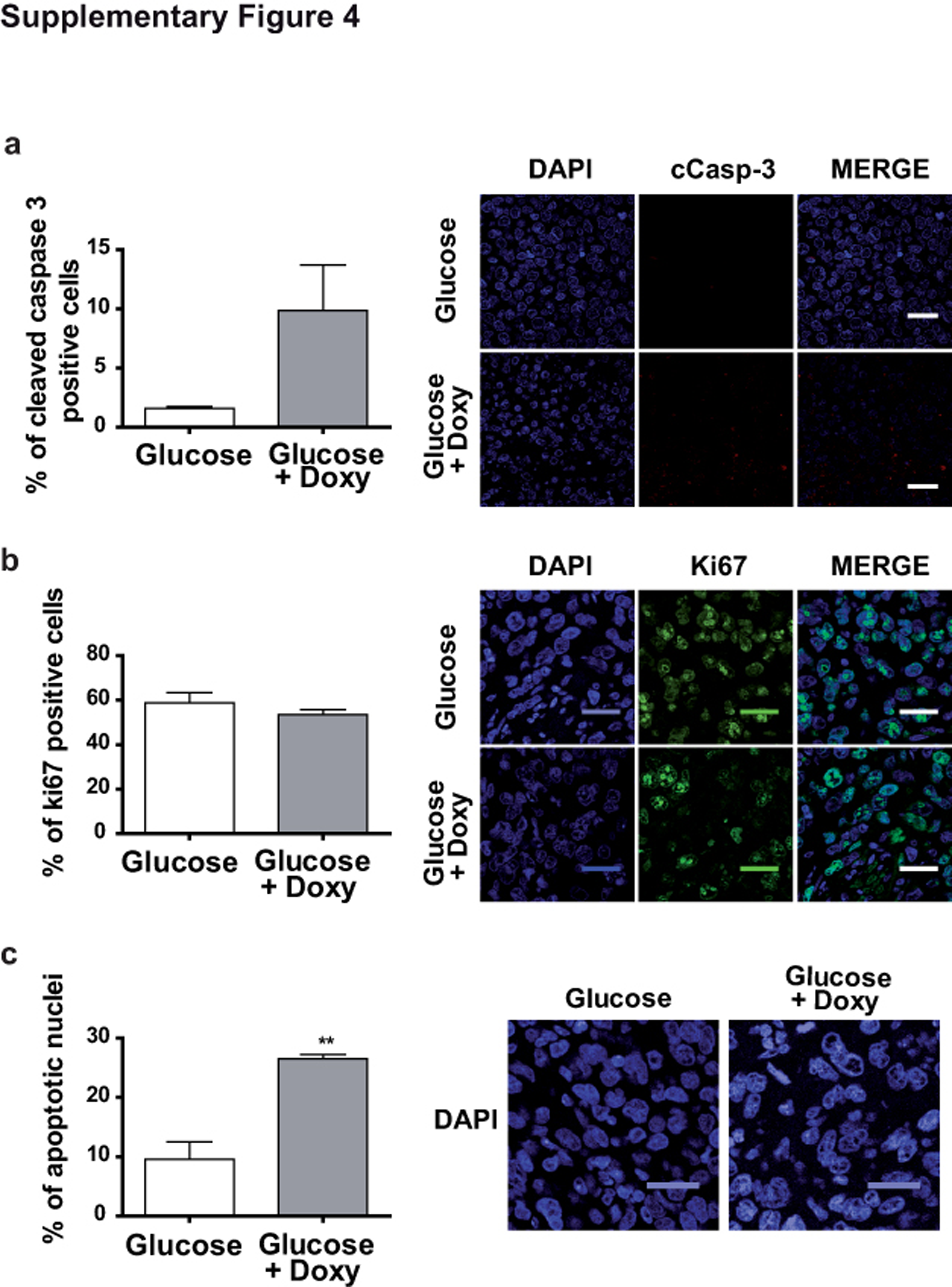

Supplement: Supplementary Figure S3 [file cddis2014594x4.tif]

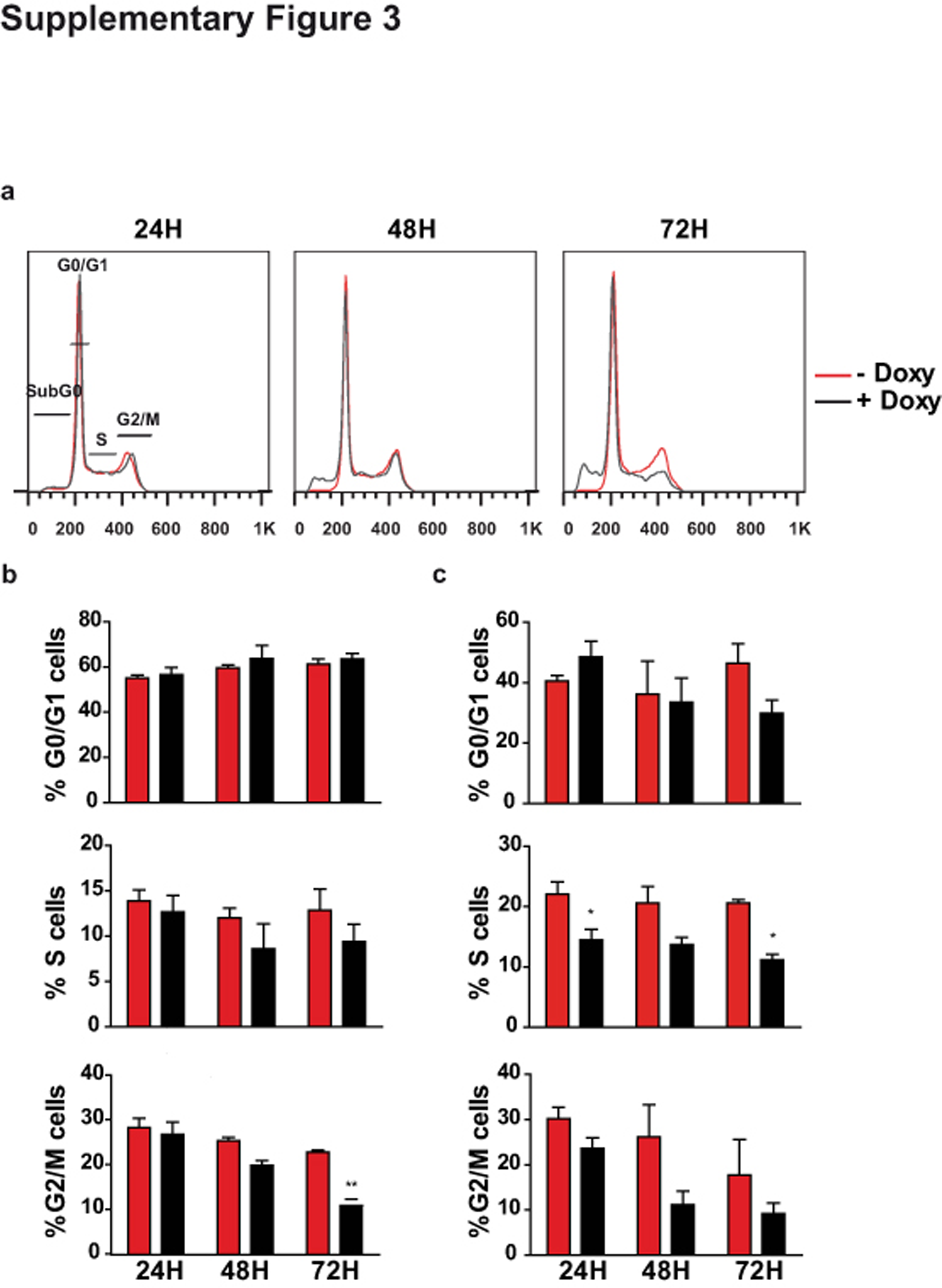

Supplement: Supplementary Figure S4 [file cddis2014594x5.tif]

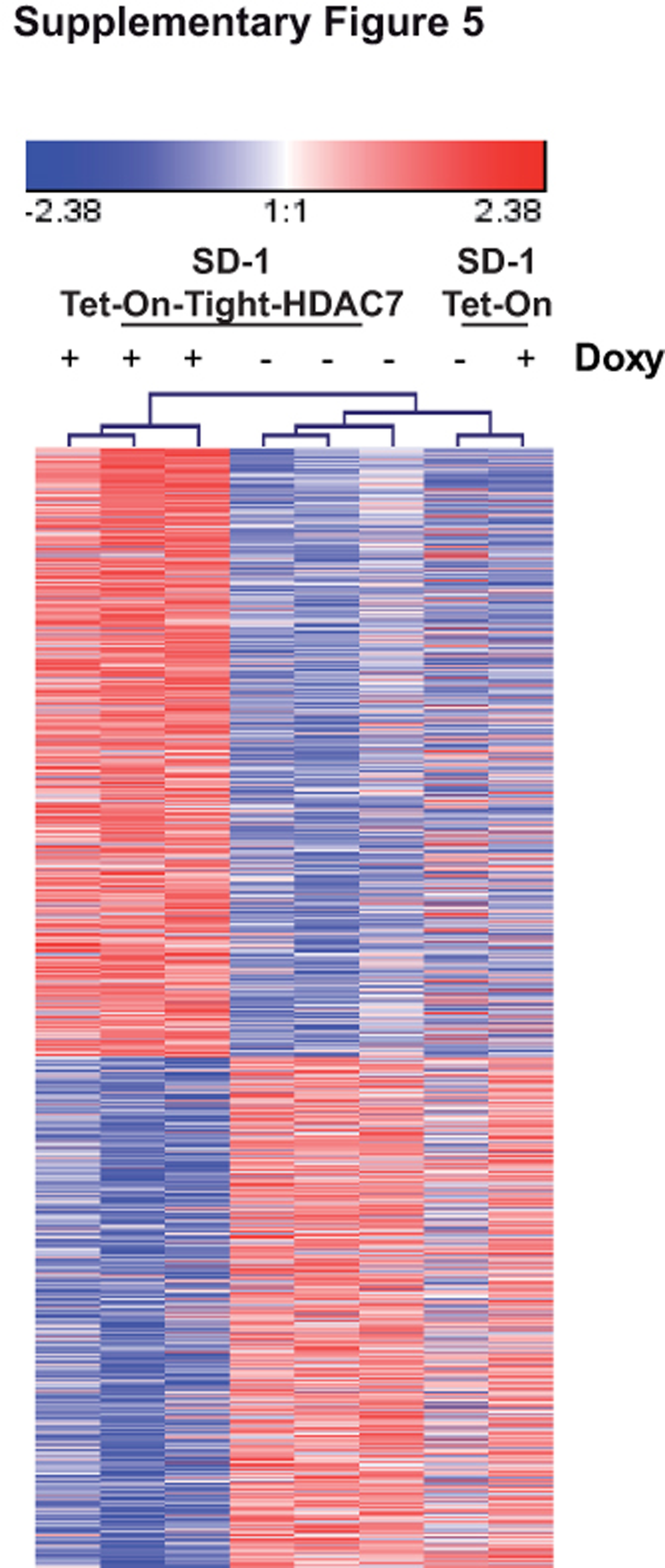

Supplement: Supplementary Figure S5 [file cddis2014594x6.tif]
