# Supplementary material for: The transcriptional repressor HDAC7 promotes apoptosis and c-Myc downregulation in particular types of leukemia and lymphoma
Source: Cell Death Dis. 2015 Feb 12;6(2):e1635–. doi: 10.1038/cddis.2014.594 (PMC4669785; doi:10.1038/cddis.2014.594)
Supplement: Supplementary Table S1 [file cddis2014594x7.doc]

**Supplementary Table S1. HDAC7 induced genes belonging to apoptosis, immune processes and** cancer categories.

| **Log2FC** | **p-Value** | **Gene Symbol** | **Apoptosis** | **Immune System** | **Cancer** | **Nature** |
| --- | --- | --- | --- | --- | --- | --- |
| 2,1 | 6,59E-03 | ANXA1 | * | * |  | Ca(2+)-dependent phospholipid binding protein |
| 2,0 | 1,30E-05 | TNFAIP2 | * |  |  | Tumor necrosis factor, alpha-induced protein |
| 2,0 | 5,05E-04 | XAF1 | * |  |  | IAP inhibitor protein |
| 1,5 | 2,72E-06 | IL16 |  | * |  | Pleiotropic cytokine |
| 1,4 | 2,42E-06 | FCGR2A |  | * |  | Immunoglobulin Fc receptor family member |
| 1,4 | 3,03E-03 | IRAK2 |  | * |  | Interleukin-1 receptor-associated kinase |
| 1,3 | 2,94E-06 | RASSF4 |  |  | * | Ras association (RalGDS/AF-6) domain family member |
| 1,3 | 5,89E-04 | RAB31 |  |  | * | Small GTP-binding proteins of the RAB family |
| 1,2 | 5,02E-05 | CR2 |  | * |  | Membrane protein |
| 1,2 | 2,29E-05 | FILIP1L |  |  | * | Filamin A interacting protein-like 1 |
| 1,1 | 8,05E-06 | GNG2 |  |  | * | Gamma subunits of a guanine nucleotide-binding protein |
| 1,0 | 1,21E-04 | NEDD9 |  |  | * | Focal adhesion protein |
| 1,0 | 1,06E-04 | CD86 |  | * |  | Antigen |
| 1,0 | 1,49E-04 | CASP7 | * |  |  | Effector caspase |
| 0,9 | 8,26E-06 | RASSF2 |  |  | * | Ras association (RalGDS/AF-6) domain family member |
| 0,9 | 1,26E-05 | BCL9L |  |  | * | nuclear cofactor |
| 0,9 | 1,48E-04 | RHOV | * |  |  | GTP binding protein |
| 0,9 | 4,79E-04 | CAPN2 |  |  | * | Calpain |
| 0,9 | 1,68E-04 | PARP14 |  |  | * | Poly (ADP-ribose) polymerase family member |
| 0,9 | 1,93E-05 | ATF3 | * |  |  | Transcription factor |
| 0,9 | 3,55E-04 | IKZF1 |  | * |  | Transcription factor |
| 0,9 | 8,03E-04 | CEACAM1 | * |  | * | Carcinoembryonic antigen (CEA) gene family member |
| 0,9 | 3,06E-04 | PLSCR1 |  |  | * | Phospholipid transfer protein |
| 0,8 | 2,93E-05 | BIRC3 | * |  |  | IAP (inhibitor of apoptosis) family member |
| 0,8 | 7,01E-05 | C1orf38 |  | * | * | Thymocyte selection assosiated family member 2 |
| 0,8 | 1,12E-03 | CD40 |  | * |  | TNF-receptor superfamily member |
| 0,8 | 5,17E-04 | SERPINA1 |  |  | * | Serine protease inhibitor |
| 0,8 | 3,83E-04 | ITGA5 |  | * | * | Intergin |
| 0,8 | 1,17E-04 | F11R |  | * | * | Immunoglobulin superfamily gene |
| 0,8 | 4,57E-05 | CCL22 |  | * |  | Cytokine |
| 0,8 | 1,36E-03 | TP53BP2 | * |  |  | Member of the ASPP (apoptosis-stimulating protein of p53) family |
| 0,8 | 3,49E-03 | SDCBP |  |  | * | Cytoskeleton regulator protein |
| 0,7 | 1,70E-04 | NCOA1 | * |  |  | Transcriptional coactivator |
| 0,7 | 8,39E-04 | CMKLR1 |  | * |  | Chemokine receptor |
| 0,7 | 1,32E-03 | ATM | * |  |  | Kinase |
| 0,7 | 2,48E-04 | MKNK2 |  |  | * | Calcium/calmodulin-dependent protein kinases (CAMK) Ser/Thr protein kinase |
| 0,7 | 2,39E-03 | TCF7 | * | * |  | Transcriptional activator |
| 0,7 | 8,92E-04 | LMNA | * |  |  | Nuclear lamina protein |
| 0,7 | 1,43E-03 | IFI44 |  | * |  | Cytoeskeleton protein |
| 0,7 | 6,79E-04 | PRMT2 | * |  |  | N-arginine methyltransferase |
| 0,7 | 3,58E-04 | SOX9 | * |  |  | Transcription factor |
| 0,7 | 3,63E-04 | SQSTM1 | * |  |  | Multifunctional protein that binds ubiquitin |
| 0,6 | 1,60E-03 | FAS | * | * |  | TNF-receptor superfamily member |
| 0,6 | 6,76E-04 | ITGB3 |  | * |  | Integrin |
| 0,6 | 3,41E-03 | RIPK1 | * | * |  | Kinase |
| 0,6 | 9,44E-04 | TNIP1 |  | * | * | A20-binding protein |
| 0,6 | 1,38E-03 | JAK1 | * |  |  | Kinase |
| 0,6 | 1,48E-03 | FCGR2B |  | * |  | Low affinity receptor for the Fc region of immunoglobulin gamma complexes |
| 0,6 | 2,06E-03 | CD44 | * | * | * | TNF-receptor superfamily member |
| 0,5 | 1,23E-03 | KDM2B | * |  |  | F-box protein |
| 0,5 | 1,67E-03 | PDE4B |  | * | * | cAMP hydrolaze |
| 0,5 | 1,41E-03 | MLH1 | * |  | * | Neoplasm protein |
