# Supplementary material for: The transcriptional repressor HDAC7 promotes apoptosis and c-Myc downregulation in particular types of leukemia and lymphoma
Source: Cell Death Dis. 2015 Feb 12;6(2):e1635–. doi: 10.1038/cddis.2014.594 (PMC4669785; doi:10.1038/cddis.2014.594)
Supplement: Supplementary Table S2 [file cddis2014594x8.pdf]

Supplementary Table S2. HDAC7 repressed genes belonging to immune processes and cancer categories.

Log2FC	pValue	Gene Symbol	Apoptosis	Immune System	Cancer	Nature
-1,5	3,41E-05	TNFRSF19			*	TNF-receptor superfamily
-1,5	8,84E-07	IGFBP3			*	insulin-like growth factor binding protein (IGFBP) family
-1,5	7,42E-05	ARHGAP18			*	Rho GTPase-activating protein
-1,4	4,12E-03	AICDA		*	*	RNA-editing deaminase
-1,2	1,90E-04	VAV3			*	Guanine nucleotide exchange factor for RHO GTPASES
-1,1	4,04E-04	ZFAT		*		Transcription factor
-1,1	2,71E-05	CERKL	*			Ceramide kinase-like protein
-1,1	4,30E-05	TERT			*	Telomerase
-1,0	1,66E-04	ITGA4		*	*	Integrin alpha subunit
-1,0	1,23E-03	MYC			*	Multifunctional nuclear phosphoprotein
-0,9	2,50E-03	GNG7			*	Guanine nucleotide-binding protein γ-7
-0,9	2,47E-04	IRF4		*	*	Lymphoid-specific interferon regulatory factor
-0,9	5,16E-04	CTSC		*		A papain-like cysteine protease
-0,8	7,52E-05	DHX33			*	DEAD box protein
-0,7	4,48E-04	MTDH			*	Cell adhesion mollecule
-0,7	1,52E-03	MYBBP1A			*	Nucleo-cytoplasmic transporter protein
-0,7	1,39E-03	EEF1E1			*	Peptide elongation factor
-0,7	5,46E-04	PNO1		*		Rna binding protein
-0,7	1,12E-03	MGLL			*	Serine hydrolase of the AB hydrolase superfamily
-0,6	2,30E-03	TNFRSF13B		*		TNF-receptor superfamily member
-0,5	2,47E-03	TPD52			*	Neoplasm protein
